# Jan Steen (c. 1625–1679). Beware of Luxury (c. 1665).

**DOI:** 10.3201/eid0908.AC0908

**Published:** 2003-08

**Authors:** Polyxeni Potter

**Affiliations:** * Centers for Disease Control and Prevention, Atlanta, Georgia, USA

**Figure:**
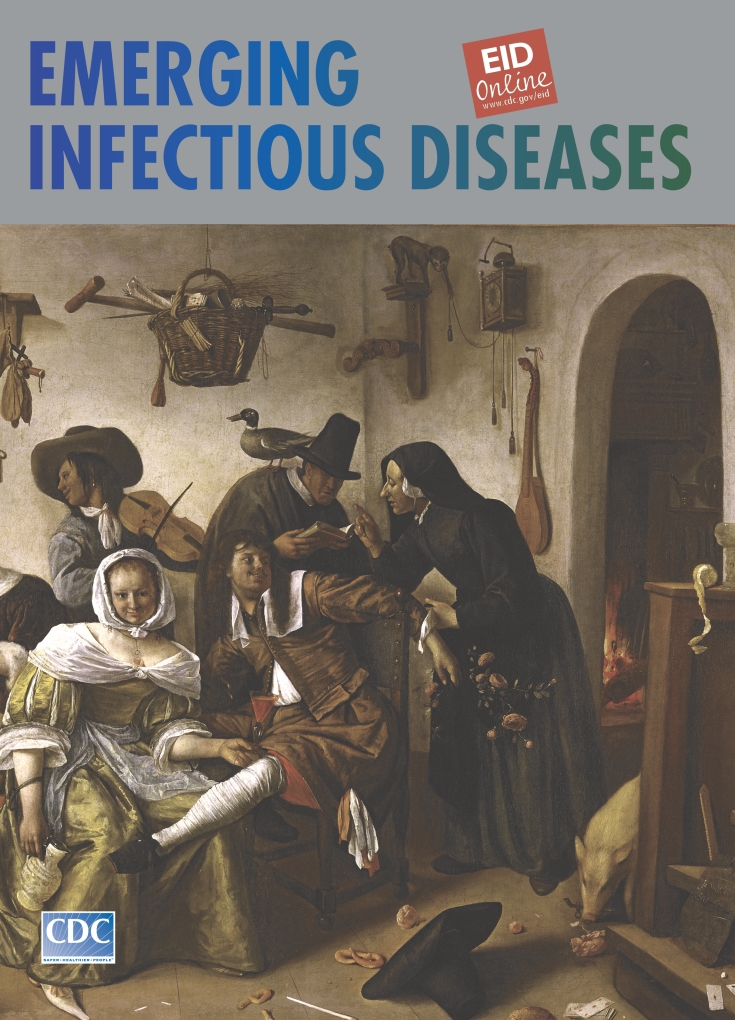
Jan Steen (c. 1625–1679). Beware of Luxury (c. 1665). Oil on canvas 105 cm x 145 cm. Kunsthistorisches Museum, Vienna, Austria

“Life is a stage; we play our part and receive our reward,” wrote Vondel, the great poet of the Netherlands, expressing the moral preoccupation of 17th-century Dutch culture (*1*). The 17th century was a time of geographic exploration and economic prosperity for the famed lowlands. Laboriously claimed from the sea, crisscrossed by canals and spotted with windmills, the tiny country produced a vibrant bourgeois society with an astonishing artistic legacy, in what has been called the Dutch golden age (*2*). Jan Steen, along with Rembrandt, Vermeer, Frans Hals, and many others who populated the local guilds, brilliantly chronicled the emergence of the distinct civilization that Oliver Cromwell said preferred “gain to Godliness” (*3*).

Jan Steen, born in Leiden the first son of successful brewers, recorded the travails and preoccupations of his contemporaries: women in their households, cavaliers on the town, village doctors, peasants in the countryside, children skating on the frozen canals, markets overflowing with goods, and the living quarters of average people. Domesticity and family harmony ruled, along with order and grace. Lavender-scented linens, Delft tiles, tended gardens, well-swept doorsteps featured in the work of Pieter de Hooch and others. But Steen, a Catholic painter in a Calvinist country, injected comic relief into the idyllic homes of the nouveau riche, by skillfully subverting the natural and social order (*2*). Genteel gatherings degenerated into drunken brawls, so much so that “Jan Steen household” became synonymous with “slovenly household” (*1*). But Steen did not condone chaos. His message, sometimes scribbled on the painting, was always clear: Memento mori (remember death).

A gifted storyteller in the tradition of Pieter Bruegel the Elder, Steen composed and embroidered with illustrated detail accounts of human behavior (*4*). Under realistic and didactic light, the shimmering garments of rich merchants and the gleaming shadows of refined interiors reflected the other side of tidy, devout Holland: crowded taverns, unruly children, untrained pets, rowdy village festivals, rampant sensuality, intemperance, and general unraveling of the domestic fabric (*2*).

Beware of Luxury, on this month’s cover of Emerging Infectious Diseases, is a humorous allegory of everyday merrymaking—celebration gone out of hand. The mother at the table near the window, in drunken stupor, is oblivious of the surroundings. The dog eats from her plate, and the children are misbehaving (smoking a pipe, raiding the cupboard, toying with mother’s pearls). A pig makes off with the spigot of the wine barrel; a monkey tinkers with the clock; a duck roosts on a guest (likely a religious quack thumbing the Bible in this ungodly context); and the man of the house succumbs to the guiles of a younger woman.

The ease and spontaneity of the painting is rivaled only by its harmonious completeness. In dead center hangs a basket prophetically filled with crutches, a bell (like the ones lepers had to carry), and a sword. Prosperity has been abused—pearls before swine. Signs of impending doom find the guilty asleep at the table. Inscribed on the right hand corner of the painting is a Dutch proverb: “In good times, beware.”

Jan Steen’s painting mirrors Dutch society of his day, which turns out not much different from our own—not only in its lack of decorum and temperance but also in its lax domesticity. In the 17th century and far before, humans cohabited with animals, on amicable terms and in close proximity. The ancient practice of keeping pets was at the heart of domestication. Exotic pets signaled power and wealth. Steen’s household would not have been complete without animals, which like children, follow in the footsteps of derelict adults—because as Steen explained in another famous painting, “You sing what you hear.”

Animals, pets and not, nest in our homes and often eat from our plates and sleep in our beds. In turn, we delight in them as companions, are awed by them in the wild, and raise them for food. This cohabitation, along with undisputed benefits, has created an evolutionary conundrum, closely involved in the emergence of infectious diseases. In some of these diseases (Ebola, hantavirus, avian flu), animals outside their natural habitat may spread viruses to humans and other species. In others (AIDS, porcine reproductive and respiratory syndrome), viruses may adapt to new hosts after unintended trans-species transmission. In yet others (severe acute respiratory syndrome, monkeypox), microorganisms, “singing what they hear,” travel from furry creatures to humans, in a complex zoonotic cycle.
